# Alterations in frontotemporal cerebral activity specific to auditory verbal hallucination during verbal fluency task in schizophrenia: a fNIRS study

**DOI:** 10.3389/fneur.2025.1559564

**Published:** 2025-06-25

**Authors:** Jiaxin Zhang, Ju Tian, Jiuju Wang, Huiting Qiao, Wenxiang Quan, Yanping Song, Daifa Wang, Wentian Dong

**Affiliations:** ^1^Key Laboratory of Biomechanics and Mechanobiology (Beihang University), Ministry of Education, Beihang University, Beijing, China; ^2^Key Laboratory of Innovation and Transformation of Advanced Medical Devices, Ministry of Industry and Information Technology, Beihang University, Beijing, China; ^3^National Medical Innovation Platform for Industry-Education Integration in Advanced Medical Devices (Interdiscipline of Medicine and Engineering), Beihang University, Beijing, China; ^4^School of Biological Science and Medical Engineering, Beihang University, Beijing, China; ^5^Peking University Sixth Hospital, Peking University Institute of Mental Health, NHC Key Laboratory of Mental Health (Peking University), National Clinical Research Center for Mental Disorders (Peking University Sixth Hospital), Beijing, China

**Keywords:** schizophrenia, auditory verbal hallucination, near-infrared spectroscopy, verbal fluency task, integral value, centroid value

## Abstract

**Background:**

Patients with auditory verbal hallucination (AVH) may experience significant occupational and social functional disabilities, which bring a heavy burden to their families and society. Although neuroimaging studies have explored the brain regions associated with AVH and proposed models to explain AVH, the potential pathological mechanisms are not clear. Functional near-infrared spectroscopy (fNIRS) is a portable and suitable measurement, particularly in exploring brain activation during related tasks. Hence, our researchers aimed to explore the differences in the cerebral hemodynamic function between patients with schizophrenia with AVH (SZ-AVHs) and patients with schizophrenia without AVH (SZ-nAVHs) through fNIRS to examine neural abnormalities associated more specifically with AVH.

**Methods:**

A 52-channel functional near-infrared spectroscopy system was used to monitor hemodynamic changes in SZ-AVHs (*n* = 178) and SZ-nAVHs (*n* = 172) during a verbal fluency task (VFT). Clinical history, and symptom severity were also noted. The original fNIRS data were analyzed using NirSpark to obtain the brain functional eigenvalues including the integral value, which represents the degree of brain activation, and the centroid value, which represents the speed of blood oxygen response.

**Results:**

Our results showed that the integral values of the SZ-AVHs were significantly higher than those of the SZ-nAVHs in the left STC [*t* = 3.16, *p* = 0.014] while the centroid values of the SZ-AVHs were significantly higher than those of the SZ-nAVHs in the right vlPFC [*t* = 2.78, *p* = 0.046].

**Discussion:**

Our findings indicate that SZ-AVHs exhibited lower activation in the left STC and Slower response speed in the right vlPFC than SZ-nAVHs.

## 1 Introduction

Auditory verbal hallucination (AVH), as one of the characteristic symptoms of Schizophrenia (SZ), is characterized as the experiences of hearing a voice or any other sounds for languages under the conditions of conscious state with no external stimulus (1). The prevalence of AVH reported rates of 60–80%, while roughly 30% of patients with AVH (2) are not responded well for current clinical treatments. As the disease progresses, patients may experience significant occupational and social functional disabilities, which bring a heavy burden to their families and society. At present, the potential pathological mechanisms are not clear, but a series of studies have explored the brain regions associated with AVH and proposed models to explain AVH.

Among them, the predictive-coding model believes that hallucination represents inner speech misattributed to an external agent due to a failure to adequately monitor and label verbal thoughts as coming from the inside rather than from the outside of the patient’s head (3). The temporal cortex is related to inner speech and the frontal cortex is related to monitoring.

Here are some studies on the relationship between the temporal cortex and AVH. A meta-analysis found that the severity of AVH is significantly correlated with a reduction in GMV in the left superior temporal gyrus (STG), and slightly correlated with a reduction in GMV in the right STG (4). In addition, the cortical thickness of the left middle temporal gyrus (MTG) has also been found to be negatively correlated with the severity of AVH in SZs. This suggests a distributional structural abnormality pattern in the temporal cortex that is specific to AVH (5). In addition to structural differences, there have also been studies comparing the differences in cerebral blood flow (CBF) between patients with schizophrenia with auditory verbal hallucination (SZ-AVHs) and patients with schizophrenia without auditory verbal hallucination (SZ-nAVHs), and found that SZ-AVHs had increased CBF in the right STG at rest (6). There are also functional magnetic resonance imaging (fMRI) studies exploring brain activation during AVH, but the result is heterogeneous (7). Some studies have found an increase in activation of the bilateral STG. But there are also studies showing significant activation of the right STG, while there is no significant activation of the left STG (8). In summary, the above studies indicate a specific relationship between the temporal cortex and AVH, but the specific brain region is still uncertain.

The frontal cortex is involved in auditory information processing and decision-making, and is also a very important brain area related to AVH. Recent studies have found that the GMV of the right ventrolateral prefrontal cortex (vlPFC) is significantly negatively correlated with the severity of AVHs (9), while the GMV of the left inferior frontal gyrus (IFG) is positively correlated with the severity of AVHs (10), indicating a specific relationship between the frontal cortex and AVH; fMRI studies investigations have revealed increased activation in the frontal cortex during AVH (7),while decreased CBF in the bilateral superior frontal gyrus (SFG) of SZ-AVHs (5), compared with SZ-nAVHs. This also indicates that the frontal cortex is involved in the occurrence of AVH, and its hemodynamic disorders may be the cause of AVH.

Currently, with the rise of functional near-infrared spectroscopy (fNIRS) which is a non-invasive imaging device to evaluate brain function, an increasing number of studies are being used to monitor changes in brain function. While the VFT is considered as the sensitive indicators of language (11) function and frontal and temporal cortex function, a large number of fNIRS studies have explored and compared the frontotemporal cortex activation between SZs and HCs by VFT. Suto found that during the VFT task, SZs had a smaller increase in oxyhemoglobin concentration (HbO) in the bilateral temporal cortex compared to HCs (12) while Tran found the same decrease in activation in the left prefrontal cortex (13). Additionally, Liang Nana explored the differences in the cerebral hemodynamic function in SZ-AVHs, SZ-nAVHs and HCs during VFT, and found that the abnormal activation in the right postcentral gyrus was correlated with severity of AVH (14). A recent study selected the integral and centroid values of brain cortical activity in the bilateral frontotemporal regions during the VFT as features for classifying SZs and HCs, with an accuracy rate of 70% (15). The above results all indicate that using fNIRS to detect brain activity during the VFT is an effective method. However, the sample size of the above studies is relatively small, and most of them only have indicators of oxyhemoglobin. Meanwhile, only a very small number of studies compare SZ-AVHs with SZ-nAVHs, lacking reproducibility, thus requiring further research.

In the present study, we utilized multi-channel fNIRS to compare the brain functional activity differences between SZ-AVHs and SZ-nAVHs during the VFT, as well as their relationship with AVH, in order to obtain AVH-specific neural mechanisms. We hypothesize that abnormal activation of the temporal lobe cortex is related to internal speech production, while functional deficits in the frontal lobe cortex may lead to decision-making errors, attributing internal speech errors to external stimuli and resulting in AVH.

## 2 Methods

### 2.1 Participants

The sample size was determined *a priori* based on power calculations for detecting between-group differences in brain activation. Assuming a medium effect size (Cohen’s *d* = 0.4) between hallucinators and non-hallucinators in fMRI activation patterns, with *α* = 0.05 (two-tailed) and 80% power, According to the formula n = 2 × (Z_1-α/2_ + Z_1-*β*_)^2^/d^2^, a minimum of 100 participants per group was required for independent samples *t*-tests. To enhance robustness against potential fMRI data variability (e.g., head motion artifacts, signal noise) and to accommodate multiple comparison correction (family-wise error rate *p* < 0.05) across whole-brain analyses, we recruited approximately 170 participants per group (total *N* = 340). This sample size provides >90% power to detect effects as small as *d* = 0.3 while allowing for covariate adjustment and exploratory subgroup analyses.

A total of 350 inpatients with Schizophrenia during Nov. 2011 to Sep. 2019 from Peking University Sixth Hospital were enrolled from inpatient ward in this study. All patients fully met the DSM-V diagnostic criteria for schizophrenia, with the following exclusion criteria: (1) alcohol or substance disorder; (2) Traumatic brain injury; (3) Neurological damage or intellectual disability. After being diagnosed and interviewed by a professional psychiatrist, the patients were divided into two groups based on whether they had auditory hallucinations: 178 patients with auditory hallucinations (SZ-AVHs) and 172 patients without auditory hallucinations (SZ-nAVHs) This study has been approved by the Ethics Committee of Peking University Sixth Hospital (Table 1).

**Table 1:** Demographic, clinical, and psychosocial characteristics of all participants [mean (SD)].

Variable	SZ-AVHs (*n* = 178)	SZ-nAVHs (*n* = 172)	χ^2^/*t/U*	*p*	Effect size (95%CI)
Gender (M/F)	101/77	109/63	1.60^a^	0.206	*V = 0.07 [0.00, 0.15]
Age ± SD	34.44 (14.72)	32.52 (12.81)	1.70^b^	0.089	*d = 0.14 [−0.06, 0.34]
Education ± SD	12.77 (3.27)	12.95 (3.59)	3.40^b^	0.638	*d = −0.05 [−0.25,0.15]
Length of illness	7.24 (8.75)	6.97 (8.29)	0.27^b^	0.790	*d = 0.03 [−0.17, 0.23]
Chlorpromazine equivalent (IQR) (mg/day)	450.00 (300.00–662.55)	399.90 (235.05–600.00)	9057.00^c^	0.196	*δ = −0.09 [−0.32,0.14]
CGI-SI	4.66 (1.02)	4.26 (1.17)	2.57^b^	0.011	*d = 0.36 [0.16, 0.56]
CGI-GI	2.43 (1.30)	2.66 (1.03)	−1.29^b^	0.202	*d = −0.19 [−0.39,0.01]
CGI-EI	7.87 (4.21)	8.02 (3.77)	−0.27^b^	0.788	*d = −0.04 [−0.24,0.16]
BPRS- illusion	3.32 (1.69)	1.39 (1.04)	8.12^b^	**<0.01**	*d = 1.36 [1.14, 1.58]
HAMD	8.21 (5.37)	8.63 (7.89)	−0.41^b^	0.686	*d = −0.06 [−0.26, 0.14]
HAMA	5.08 (3.83)	5.11 (5.64)	0.05^b^	0.961	*d = −0.01 [−0.21, 0.19]

IQR, Interquartile Range; CGI, Clinical Efficacy Inventory; CGI-SI, Clinical Global Impression-Severity of Illness; CGI-GI, Clinical Global Impression-Global Improvement; CGI-EI, Clinical Global Impression-Efficacy Index; BPRS- Illusion, Brief Psychiatric Rating Scale Illusion; HAMD, Hamilton Depression Scale; HAMA, Hamilton Anxiety Scale; ^a^χ^2^ test; ^b^ t test; ^c^ a Mann–Whitney U test; *V: Cramer’s V; *d: Cohen’s d; *δ: Cliff ‘s Delta; 95% CI: 95% confidence interval. Bolded values represent *p* < 0.01 statistical significance.

The relevant scales will be evaluated by well-trained psychiatrists less than 1 week before fNIRS measurement. The overall clinical status and treatment response of patients were evaluated using the Clinical Efficacy Inventory (CGI), which focuses on disease severity (CGI-SI), overall improvement (CGI-GI), and treatment efficacy index (CGI-EI); The Hamilton Depression Rating Scale (HAMD) and Hamilton Anxiety Rating Scale (HAMA) are used to assess the severity of depression and anxiety. The Brief Psychiatric Rating Scale (BPRS) is used to assess the severity of the disease, and the severity of AVH is quantified using the Brief Psychiatric Rating Scale Illusion (BPRS- Illusion) score. Medication dosages are presented as chlorpromazine-equivalent doses calculated using the DDD conversion factors (WHO Collaborating Centre for Drug Statistics Methodology); All participants are native Chinese speakers and able to read Chinese.

### 2.2 Verbal fluency task

We adopted a Chinese version of phonological VFT. This measurement was taken under a quiet environment. Participants were asked to remain seated with their eyes open, avoid excessive body, minimize head movements, and focus on a cross-displayed during the measurements. It comprised a 30-s pre-task period, a 60-s task period, and a 70-s post-task period. During the pre- and post-task periods, the participants were asked to constantly say “1, 2, 3, 4, 5” repeatedly. During the task period, the participants were asked to generate as many four-character idioms or phrases as possible, which begin with the designated Chinese characters (such as, “大,” “白,” and “天,”) indicating big, white, and sky, respectively. There was a total of three cue characters that were changed every 20 s during the 60-s task period.

### 2.3 NIRS measurement

A 52-channel fNIRS system (ETG-4100. Hitachi Medical Co., Tokyo, Japan) uses 2 NIR light wavelengths (695 and 830 nm) to measure the hemodynamic responses in the prefrontal cortices and superior temporal cortices. This system had 16 light detectors and 17 light emitters, all of which were arranged in a 3 × 11 array to form 52 measurement channels according to the international 10–20 system. This arrangement allowed for hemodynamic response covered mainly in the entire bilateral prefrontal cortices, and the anterior and superior parts of the temporal cortex to be measured.

### 2.4 NIRS data analysis

We used the NirSpark software package (16) to analyze NIRS data. Data were preprocessed via the following steps. Motion artifacts were corrected by a moving SD and a cubic spline interpolation method. A bandpass filter with cut-off frequencies of 0.01–0.20 Hz was used for resting state data and a 0.2 Hz low-pass filter was used for VFT state data to remove physiological noise (e.g., respiration, cardiac activity, and low-frequency signal drift). The modified Beer- Lambert law was used to convert optical densities into changes in the deoxyhemoglobin and oxyhemoglobin concentrations.

Integral and centroid values of the Regions of Interest (ROIs) during VFT were generated from the NirSpark (17) by evaluating the hemodynamic changes in the concentrations of oxyhemoglobin (HbO), deoxyhemoglobin (HbR), and total hemoglobin (HbT) of the 10-s pre-task,60-s task, and 55-s post-task period from the original 160-s VFTs. And the frontotemporal region was segmented into eight ROIs: (1) right dorsolateral prefrontal cortex (dlPFC); (2) dorsal frontopolar cortex (dFPC); (3) left dlPFC; (4) right superior temporal cortex (STC); (5) right ventrolateral prefrontal cortex (vlPFC); (6) ventral FPC (vFPC); (7) left vlPFC; and (8) left STC. Integral value, measured in mmol/L * mm * s, was calculated using the hemodynamic response of HbO, HbR and HbT during the 60-s activation task period by averaging the signal from channels within each region (18); The centroid value is an index of time-course changes throughout the VFT, which refers to the point in the waveform where the hemoglobin positive direction reaches half of the integral value, with periods representing the timing of the hemodynamic response.

### 2.5 Statistical analysis

SPSS (Statistical Package for the Social Sciences for Windows, version 26; IBM SPSS Statistics, New York, United States) was used to compare basic information and clinical data in each group. For categorical variables (presented as frequencies), between-group differences were assessed using χ^2^ tests, with effect sizes reported as Cramer’s V and 95% confidence intervals. Continuous variables (e.g., age, education, illness duration, and scale scores) (presented as mean ± SD) were compared using independent samples *t*-tests, with effect sizes reported as Cohen’s d and corresponding 95% confidence intervals. Non-normally distributed olanzapine-equivalent [presented as median (IQR)] were compared using a Mann–Whitney U test, with effect sizes reported as Cliff’s Delta and corresponding 95% confidence intervals (15). The brain functional eigenvalues between the two groups were compared using the *t*-test, with effect sizes reported as Cohen’s d and corresponding 95% confidence intervals, and Pearson’s correlation coefficient was used to determine the relationship between brain functional activity and the severity of AVH. For multiple comparisons of multiple channels, False Discovery Rate (FDR) correction method is used to correct a class of error probabilities. *p*(FDR) < 0.05 was considered statistically significant.

## 3 Results

### 3.1 Demographic and clinical characteristics

The basic information and clinical data of the participants are shown in Table 1. There were no significant differences in gender, age, education, disease duration, as well as HAMD and HAMA scores, CGI-GI and CGI-EI scores between the two groups of participants. The CGI-SI scores showed a small but significant effect (*d* = 0.36, 95% CI [0.16, 0.56]), indicating slightly greater illness severity in the SZ-AVHs group. Most notably, hallucination scores demonstrated a large and highly significant effect (*d* = 1.36, 95% CI [1.14, 1.58]), reflecting substantially more severe psychotic symptoms in SZ-AVHs patients.

### 3.2 The brain functional eigenvalues in ROI

**Figure 1:**
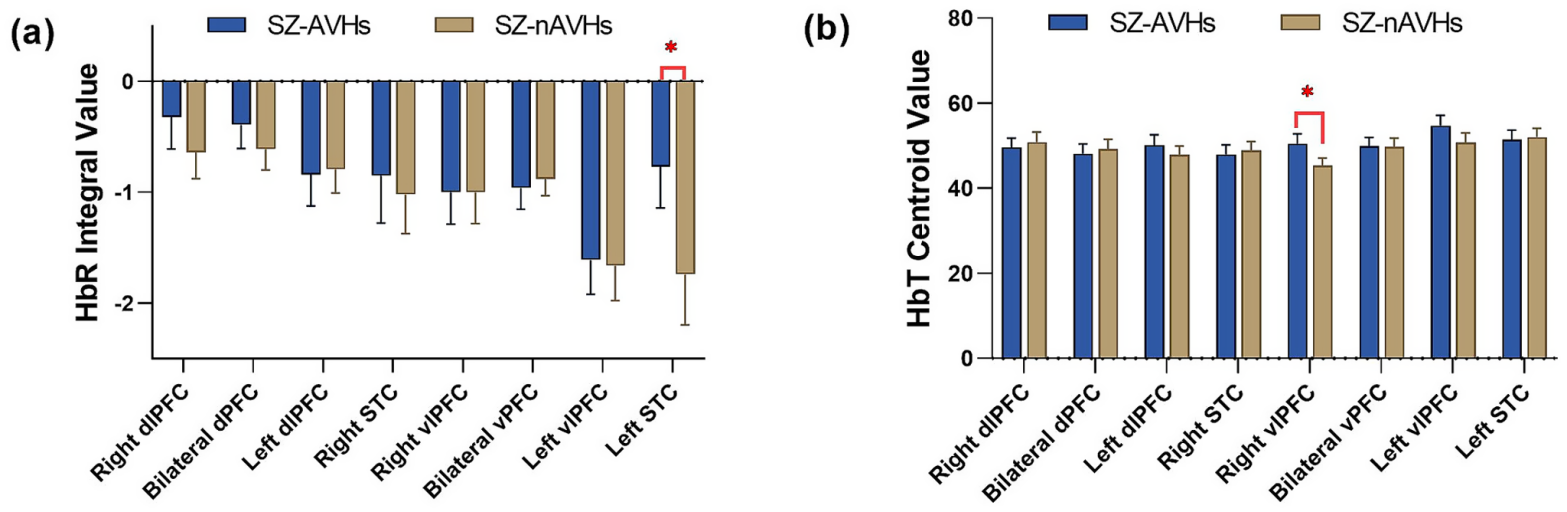
Comparison of frontotemporal integral and centroid value of fNIRS signals between SZ-AVHs and SZ-nAVHs. **(a)** The integral values of HbR during the VFT period. **(b)** The centroid values of HbT during the VFT period; Error bars represent standard error of measurement, *p < 0.05.

After FDR correction, the integral values of the SZ-AVHs were significantly higher than those of the SZ-nAVHs in the left STC [*t* = 3.16, *p* = 0.014, *d* = 0.20, 95% CI [0.04, 0.36]] while the centroid values of the SZ-AVHs were significantly higher than those of the SZ-nAVHs in the right vlPFC [*t* = 2.78, *p* = 0.046, *d* = 0.21, 95% CI [0.05, 0.37]] (Figure 1).

**Figure 2:**
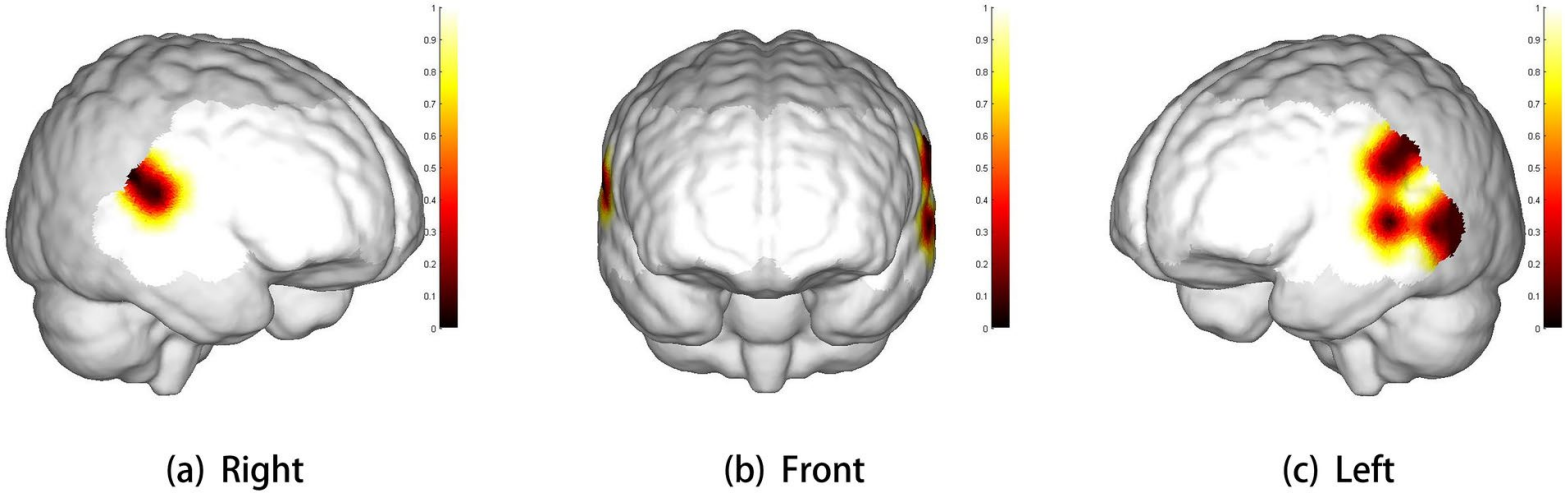
Three-dimensional cerebral maps of significant differences between two groups. The scale bar represents *p*-value. White represents the coverage of brain regions (*p* > 0.1). Panels **(a–c)** show the right view, front view, and left view, respectively.

The three-dimensional topographic map of brain regions with significant differences in brain activation levels is shown in Figure 2.

### 3.3 Correlation between demographic characters and fNIRS variables

For the left STC with significant differences in integral values after FDR correction, Pearson correlation analysis was performed on the integral values of all channels in the region and their corresponding BPRS Illusion scores, with CGI-SI scores as the control variable. The BPRS- Illusion scores was found to be significantly correlated with the HbR integral values of channel 33 (*R* = 0.140, *p* = 0.018), while there was no significant correlation with other channels.

## 4 Discussion

This study used fNIRS to explore the association between hemodynamic response in the frontotemporal cortex and AVH in patients with SZ. Our findings indicate that SZ-AVHs exhibited lower activation in the left STC and Slower response speed in the right vlPFC than SZ-nAVHs.

First, in terms of HbR, our study observed that the SZ-AVHs had lower brain activation in left STC in contrast to the SZ-nAVHs, which were similar to the previous fNIRS studies showing the lower brain activation in similar brain cortices during the related task (19), But for HbO, there was no significant difference, which may indicate that HbR is more sensitive than HbO in this study. This speculation was predicted from the theoretical grounds of the BOLD response and is in agreement with several previous works (20). The consistency between the fNIRS results in this study and the fMRI research results also indicates that fNIRS is a suitable research tool to explore the underlying neural alterations of patients with AVH during related tasks (21). However, other studies have reported contradictory results, suggesting that HbO may be a better indicator than HbR. This may be due to the long task time, which cannot reflect the advantage of HbR’s fast response speed, and the high signal-to-noise ratio of HbO measurement (17). In all, there is still controversy over which of the three concentrations of HbO, HbR, and HbT is more sensitive. There have also been studies on patients with mental disorders such as depression using VFT through fNIRS, and it was found that their corresponding brain regions were activated. There are also studies exploring the changes in brain activity of mental disorders such as depression during VFT using fNIRS (12), and finding that their corresponding brain regions produce similar activations. There was no significant difference in HAMA and HAMD scores between the two groups in this study, indicating that the results were not affected by depression or anxiety.

We also detected changes in frontal cortex blood flow during VFT in patients with and without AVH, and found that SZ-AVHs had a significant delay in reaching the peak of HbT in the right vlPFC, This is similar to a previous study that distinguished schizophrenia from other mental disorders by centroid values, with an accuracy rate of up to 84% (22). This indicates that SZ-AVHs have significant deficits in frontal lobe function (23), which is related to semantic control (24). A study suggested that disruption to medial anterior PFC, may be at least partly responsible for an impairment in making similar discriminations that might account for the hallucinations associated with schizophrenia (25). However, there was also a significant difference in the severity of the disease between the two groups, so slower frontal cortex response may also be related to the severity of the disease.

The above neuroimaging results can also provide an explanation for the predictive-coding model. The predictive coding model states that AVH in SZ is associated with increased baseline activity in the associative auditory cortex (3), such as temporal cortex. But when internal speech is perceived by the same receptors, the corresponding brain regions will be inhibited, and the degree of inhibition will be lower compared to healthy individuals (26). In this study, SZ-AVHs activation was reduced compared to SZ-nAVHs. The above also indicates that functional defects in the temporal cortex can lead to auditory hallucinations. The slower response speed of the frontal lobe also indicates hemodynamic disorders and functional decline in this area, leading to dysregulation of monitoring ability (27) and causing internal speech to be mistakenly attributed to external stimuli, resulting in auditory hallucinations.

Nevertheless, there are still several limitations associated with our research. Firstly, the coverage of brain regions is limited, with only the frontotemporal cortex being studied and no mention of brain activity related to other areas. Second, the scale used to assess auditory hallucinations is not the most commonly employed measure in clinical practice, which may limit the accuracy of our hallucination evaluations. This could contribute to the modest correlation coefficients observed (Table 2), potentially reducing their clinical applicability. Meanwhile, the differences in disease severity (as indicated by CGI-SI scores) between the two groups also preclude the certainty that the study findings are specifically attributable to the effects of AVH. Further research is required to control for disease severity in order to clarify this issue. And there is not the information of behavioral performance on the VFT, which could differ between groups and impact neural activation patterns. Finally, although there was no significant difference in olanzapine equivalent doses between the two groups, the potential impact of different medication types and unknown drug–drug interactions on the outcomes cannot be ruled out. Future studies should control for specific antipsychotic agents and their dosages with standardized medication protocols to clarify these effects. Despite these challenges, our research findings still provide additional support for the identification of brain region biomarkers of AVH and offer more possibilities for the subsequent treatment of AVH (28). We also believe that future research and technological innovation will help overcome these limitations, and provide more evidences for the common theories regarding the AVH.

**Table 2:** Correlation analysis between HbR integrated values of left STC and BPRS- Illusion scores.

Ch	Pearson correlation	
	*R*	*p*
Ch31	0.039	0.649
Ch41	0.140	0.018*
Ch42	0.041	0.489
Ch51	0.102	0.086
Ch52	0.061	0.300

*p < 0.05.

## Data Availability

The raw data supporting the conclusions of this article will be made available by the authors, without undue reservation.
